# Altering social cue perception impacts honey bee aggression with minimal impacts on aggression-related brain gene expression

**DOI:** 10.1038/s41598-019-51223-8

**Published:** 2019-10-10

**Authors:** James W. Harrison, Joseph H. Palmer, Clare C. Rittschof

**Affiliations:** 1 Department of Entomology, University of Kentucky, S-225 Agricultural Science Center North, Lexington, KY 40546 USA; 2 College of Agriculture, Communities, and the Environment, Kentucky State University, 400 E. Main St., Frankfort, KY 40601 USA

**Keywords:** Molecular neuroscience, Animal behaviour

## Abstract

Gene expression changes resulting from social interactions may give rise to long term behavioral change, or simply reflect the activity of neural circuitry associated with behavioral expression. In honey bees, social cues broadly modulate aggressive behavior and brain gene expression. Previous studies suggest that expression changes are limited to contexts in which social cues give rise to stable, relatively long-term changes in behavior. Here we use a traditional beekeeping approach that inhibits aggression, smoke exposure, to deprive individuals of aggression-inducing olfactory cues and evaluate whether behavioral changes occur in absence of expression variation in a set of four biomarker genes (*drat*, *cyp6g1/2*, *GB53860*, *inos*) associated with aggression in previous studies. We also evaluate two markers of a brain hypoxic response (*hif1α*, *hsf*) to determine whether smoke induces molecular changes at all. We find that bees with blocked sensory perception as a result of smoke exposure show a strong, temporary inhibition of aggression relative to bees allowed to perceive normal social cues. However, blocking sensory perception had minimal impacts on aggression-relevant gene expression, althought it did induce a hypoxic molecular response in the brain. Results suggest that certain genes differentiate social cue-induced changes in aggression from long-term modulation of this phenotype.

## Introduction

Genomics approaches have transformed behavioral ecology, providing new insights into the molecular and genetic basis of complex, naturally occurring phenotypes in non-model organisms^1–11^. One common but somewhat surprising pattern in this field is that exposure to a brief social cue causes extensive changes in brain gene expression. This pattern is observed in species as diverse as insects, fish, and mammals^12–14^. The genes that show dynamic expression in response to a social interaction are often assumed to play an important role in regulating behaviors that are expressed in response to social contact (e.g., aggression, care behavior, and or courtship interactions)^2,15^. Moreover, gene expression changes following a brief social interaction are thought to underlie persistent changes in behavioral phenotype^8^. However, few studies have directly evaluated how gene expression patterns scale to physiological processes in the brain that regulate behavioral expression. As a result, the implications of the broad molecular response to social information persists as a substantial knowledge gap in behavioral genomics. Knowing the role of gene expression dynamics in regulating behavioral phenotypes is critical to understanding the expression of social behaviors, which are known for their exceptional degree of plasticity^16^.

While socially-induced gene expression changes may lead to long-term behavioral consequences in some cases, it is also possible that these molecular dynamics simply reflect the activity and recovery of neural circuitry associated with sensory perception and immediate behavioral response^8,15,17^; a neural signaling event often leads to a temporary change in mRNA abundance known as the “genomic action potential”^18^. There are many approaches that could be used to distinguish these alternative possibilities. One is to evaluate whether brain gene expression changes are limited to social contexts that evoke long-lasting changes in behavior. This approach is challenging, however, due to the range of possible timescales relevant to behavioral plasticity, and the vast number of genes modulated by a social interaction (which may have diverse functions). Another approach is to specifically modulate an individual’s sensory capabilities and determine the degree to which a change in sensory signaling is responsible for variation in brain gene expression. In the current study, we employ the latter approach in the context of honey bee (*Apis mellifera*) aggression.

An extensive body of literature has assessed the relationship between socially modulated aggressive behavior and brain gene expression in the honey bee (reviewed in ref.^8^). As such, this system provides a model to study how molecular changes in the brain give rise to behavioral expression in a social context^14^. Aggression is displayed by worker bees defending the hive against large mammalian predators or intruding foreign bees from neighboring hives^19–21^. Repelling a predator requires the coordinated action of hundreds or even thousands of individual workers^19^. To facilitate this, individual aggression is highly responsive to social information: a worker bee’s developmental environment impacts her responsiveness to aggressive cues once she becomes an adult^22^, and the phenotypes displayed by nestmates further modulate individual aggression throughout adulthood^23–25^. Repeated colony-wide exposure to predator threat and its associated social cues can either increase^17,26^ or decrease^9^ aggression depending on the intensity of the stimulus. All of these contexts for aggressive response are associated with changes in brain gene expression (typically measured at the whole brain level). For example, exposure to a 1-min alarm olfactory cue is sufficient to cause a change in expression for hundreds of genes in the honey bee brain^17,23,27^.

In a recent study, we found that social context can temporarily modulate individual honey bee aggression without changes in brain gene expression^28^. This finding, which contradicts most previous work, suggests that a neuromolecular change is not required for an easily reversed, socially-mediated aggressive response. We thus hypothesize that social cue perception alone is not sufficient to cause changes in brain gene expression, and moreover, that such gene expression changes are limited to contexts of long-lasting behavioral modulation^17,26^. We explicitly test these ideas using a unique experimental approach in the honey bee to examine how social cue perception influences behavior and brain gene expression. We use a common beekeeping practice, smoke exposure, to block perception of aggression-inducing social cues and temporarily inhibit aggressive behavior^29^. We compare behavioral and brain gene expression dynamics to a second group that is allowed to perceive typical aggression-inducing social cues and mount a defensive response. We hypothesize that bees allowed to perceive aggression-inducing social cues will exhibit a more aggressive phenotype compared to those deprived of social cues using smoke. However, we also predict few aggression-related changes in the brain molecular state as a result of social cue exposure, due to short-lived behavioral effects, and manipulation of sensory perception alone^9,23,28^. To assess aggression-related gene expression, we use a set of four biomarker genes (*drat*, *cyp6g1/2*, *GB53860*, *inos*) whose expression levels robustly track socially-induced variation in aggression in a range of contexts in previous studies^9,28^. We contrast biomarker expression with two markers of hypoxia (*hif1α*, *hsf*) to determine whether smoke has a molecular signature in the brain that may serve purposes other than the regulation of aggressive behavior.

## Methods

### Timeline for data collection

During summer 2017, we collected aggression data comparing smoke-exposed (3 min post-exposure) and an unexposed control, as well as gene expression data for smoke-exposed bees (3 min and 10 min post-exposure). During summer 2018, we repeated the behavioral data collection for additional time points: control, 3-min, 10-min, and 30-min post exposure.

### Bee collection and preparation

Honey bees (*Apis mellifera*) used in this study were a mixture of genetic backgrounds, including Carniolan, Italian, and Russian hybrids, maintained on the University of Kentucky campus according to standard beekeeping practices and disease management recommended by the Honey Bee Health Coalition. We collected adult forager honey bees to use in the behavioral experiments from a total of four colonies. Gene expression data represent collections from two different colonies. Following previous studies^22,28,30,31^, returning foragers were vacuumed at the entrance of the hive and immediately transferred to assay arenas (100 mm × 20 mm petri dishes, 6 bees per dish). Bees were kept in groups because the assay of aggression described below requires a social context. We provisioned bees *ad libitum* with 50% sucrose (m/v) dispensed with two 1.5 mL centrifuge tubes with holes drilled in the base. Each assay arena had three circular openings on its lid: one ~1 cm wide opening for intruder bee introduction (see below), one ~4 mm wide opening for the syringe and smoke application (see below), and one ~1 cm wide opening for smoke ventilation. The ventilation opening was covered with 1 mm fiberglass screen, and the other two openings were sealed with tape. We left bees in a laboratory fume hood overnight (25 °C) prior to initiating the experiment.

### Smoke treatments

We produced smoke in an outdoor area adjacent to the laboratory with a typical honey bee smoker (Pro-Bellow Smoker, Mann Lake Limited, Hackensack, MN, USA). Following standard practice, we packed the cylinder with cedar wood shavings and a small amount of paper, and ensured the smoker was well-lit and generating a thick smoke plume prior to initiating the experiment. The smoke was collected at the base of the plume using a 10-mL syringe (Walmart Incorporated, Bentonville, AR, USA). To ensure uniformity, the smoke in the syringe was only used if it was opaque and gray-colored. Filled syringes were sealed with a square of paraffin film, then brought to the location of the assay arenas and administered immediately (transfer time ~1.5 min).

The groups of bees were assigned randomly to treatments. For the control group, 10 mL of ambient air was injected into the arena 3 min prior to performing behavioral assays (detailed below). For the smoke treatments, we injected 10 mL of smoke and then waited 3, 10, or 30 min prior to flash freezing bees for molecular analyses or performing behavioral assays (see below). After injection, the smoke diffused rapidly out of the arena through the mesh opening. In 2017, experiments were performed in a laboratory fume hood. We set up control, 3-min smoke, and 10-min smoke treatments, but measured behavior for the control and 3-min smoke treatments only. For these two groups, bees were flash-frozen immediately after the 2-min behavioral assay (see below). For the 10-min smoke treatments, bees were immediately flash frozen with liquid nitrogen 10 min following smoke injection. Frozen samples were stored at −80 °C for later gene expression analysis. We performed 2018 experiments outside and did not retain any samples for gene expression analysis.

### Aggression assays

Behavioral assays followed previously published methods^22,28,30^. Briefly, we collected “intruder bees” (returning forager bees vacuumed at the colony entrance) from a colony independent from the others used for the experimental groups. Introduction of a foreign bee into a group of nestmates provokes aggression from the group members. Group members use odor cues to distinguish nestmates from foreign bees^32^. At the designated time point, we introduced an intruder bee marked on the thorax with paint (Testors, Rockford, IL, USA) into the petri dish through the 1 cm opening. We tallied aggressive behaviors displayed by group members, including antennation, antennation with mandibles open, biting, abdomen flexion, and stinging over two minutes. These tallies were used to calculate an aggression score for each group of bees. This score weights behaviors for severity, with actions like biting and stinging the intruder scoring more points than low-level aggressive actions like antennation^33^. We report total scores in addition to tallies from individual behaviors.

### Brain gene expression analyses

To evaluate whether smoke-induced variation in aggression is accompanied by shifts in brain molecular state, we used qPCR to measure whole-brain gene expression levels for four aggression biomarker genes^9,28^. Previous studies show that the whole-brain expression levels of these genes are strongly correlated with variation in aggression, whether that variation is a function of age, genotype, or short or long term social experience^9,23,28^. In addition to these genes, we measured *hif1α* and *hsf*, genes canonically associated with the neural protective response to hypoxia^34,35^. *Hsf* is a downstream target of *hif1α*, and it is required for activation of heat shock protein response to hypoxia, an essential response for retaining viability under low oxygen conditions^36^. We measured two endogenous control genes (*eIF3-S8* and *rpS5a*) previously shown to have limited expression variation in brain tissue of forager-aged bees differing in aggression levels^9^. All targets and their primer sequences are listed in Table 1. Bee heads were partially freeze-dried at −90 °C for 22–28 min (Labconco, Kansas City, MO, USA) and dissected in 95% ethanol on dry ice^9^. We extracted mRNA using an E.Z.N.A. HP Total RNA Kit (Omega Bio-tek, Norcross, GA, USA) with an on-column DNAase treatment (Omega Bio-tek). We synthesized cDNA from 200 ng RNA using a SensiFAST™ cDNA Synthesis Kit (Bioline, Taunton, MA, USA) and performed qPCR on a Quanta Studio 6 (ThermoFisher) with 10 µL reactions (in triplicate on a 384-well plate) using PerfeCTa SYBR Green SuperMix (Quantabio, Beverly, MA, USA).

**Table 1:** qPCR targets and primers.

Name	NCBI	BeeBase ID	Forward	Reverse
*eIF3-S8*	551184	GB41874	TGAGTGTCTGCTATGGATTGCAA	TCGCGGCTCGTGGTAAA
*rpS5a*	409728	GB45730	AATTATTTGGTCGCTGGAATTG	TAACGTCCAGCAGAATGTGGTA
*drat*	408643	GB55016	CACGACATCACGCAGCCTT	CCTTGATCACGAACACCACG
*inos*	551143	GB51125	CCATTAGTACCACGAGGAACGC	TTTCAATTGCAGCTCGTTGTCT
*cyp6g1*/*2*	408383	GB52023	TTCGCAAATATCCACCTCTAGGA	TCTATCGTCACATCAGAATTGGGT
*Unknown*	724644	GB53860	GAACGCAAAGAGCAACCGA	CCATCCGCAGGGTGTATCATA
*hif1α*	408852	GB44532	TTTTGCACGGAGGAACGATG	AATACCCGTTGCCGAAATCC
*hsf*	411854	GB52628	TGCAATGGAGCAAGCACAAG	TGTAAGCAGCGTCACACATG

### Statistical analyses

We performed statistical analyses with JMP Pro 12 (SAS Institute, Cary, NC). We log-transformed total aggression scores for normality, and analyzed behavioral and gene expression data using parametric statistics.

## Results

**Figure 1:**
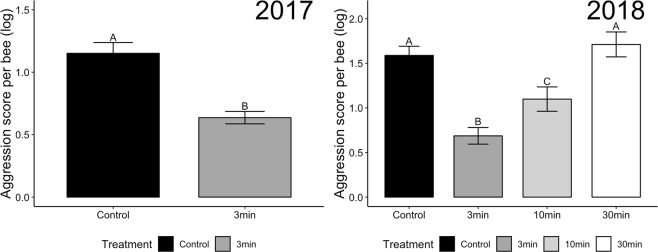
Smoke exposure significantly decreased aggression. In 2017, we measured aggression for unexposed control bees and bees 3 min following smoke exposure. In 2018, we measured unexposed control bees and bees 3 min, 10 min, and 30 min following smoke exposure. In both years, smoke significantly decreased aggression after 3 min, and in 2018, this decrease persisted for the 10 min time point (bees regained typical aggression after 30 min). Letters represent groups that are significantly different, as identified using a t-test (2017) and an ANOVA followed by a post-hoc T-test of all pairs (2018). The aggression score is a cumulative value that weights instances of aggressive behaviors by severity.

**Figure 2:**
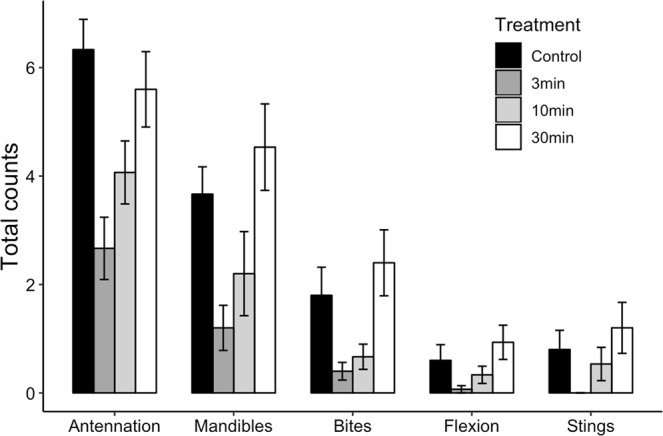
Smoke exposure affects aggressive behaviors that range in intensity. The temporal dynamics of the change in aggression over time following smoke exposure was similar across a range of aggressive behaviors. Data are represented as the mean and S.E. of total counts of observations of aggressive behaviors per group of bees. Behaviors are ordered in terms of intensity (antennation is the least intense, stinging is most intense).

In both years of data collection, smoke treatment rapidly and robustly decreased aggression (2017: two-tailed T-test, t_18_ = −5.1, P < 0.0001; 2018: ANOVA, F_3,56_ = 15.5, P < 0.000, Fig. 1). For the larger 2018 dataset, post-hoc T-tests across all treatment pairs showed that smoke exposure decreased aggression significantly relative to control at 3 min. At 10 min, aggression remained lower than the control, but increased significantly compared to the 3-min time point. By the 30-min time point, aggression returned to control levels. Looking across different types of aggressive behaviors ranging in severity from antennation to stinging, the pattern associated with smoke exposure over time was consistent (Fig. 2).

**Figure 3:**
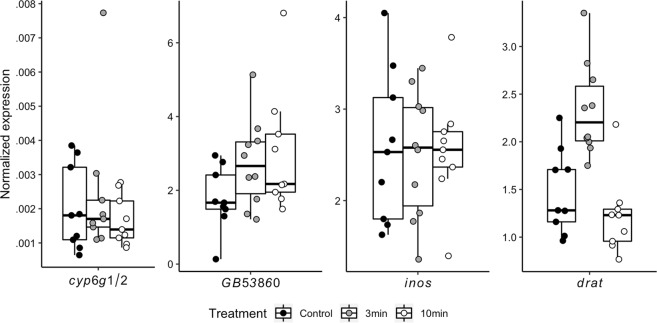
Smoke exposure had minimal impacts on expression of aggression biomarker genes. Gene expression was evaluated using whole-brain samples and normalized to an endogenous control gene. Only *drat* shows significant variation as a function of aggression (P < 0.0001), but the direction of change is the opposite of the typical relationship to low aggression. Statistical details are shown in Table 2.

**Figure 4:**
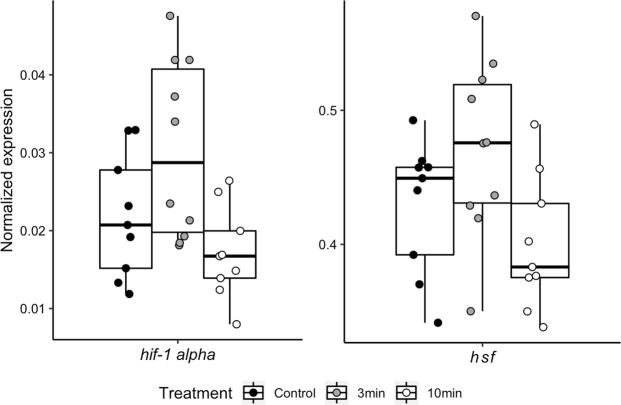
Smoke exposure caused expression differences for two hypoxia-related genes. Gene expression was evaluated using whole-brain samples and normalized to an endogenous control gene. Both genes showed increased expression at the 3-min time point followed by a decrease in expression back to (*hif1α*) or below (*hsf*) control levels by the 10-min time point. Statistical details are shown in Table 2.

In our brain gene expression analyses (N = 9 for Control, N = 10 for 3 min, N = 9 for 10 min, chosen haphazardly from the larger set of bees), *eIF3-S8* and *rps5a* both had low (<22%) coefficients of variation, but *eIF3-S8* showed significant differences among treatments (F_2,50_ = 3.68, P = 0.032), and so it was excluded from use as a control gene. All target genes were normalized to *rps5a*. One sample was removed from the analysis of *cyp6g1/2* because it was more than three standard deviations above the mean. Of the four aggression biomarker genes, only one, *drat*, was differentially expressed as a function of smoke exposure (Fig. 3, Table 1). Expression increased at 3 min, but then decreased to control levels at 10 min. Both hypoxia genes showed evidence of differential expression (Fig. 4). *Hif1α* increased at 3 min and then returned to control levels. *Hsf* increased at 3 min and then dipped significantly below control expression levels at the 10-min time point.

**Table 2:** Gene expression analyses (ANOVA followed by post-hoc each pairs T-tests for significant results). The same data were used to produce Figs 3 and 4.

Name	BeeBase ID	Relevant context	Description	F	P	Post-hoc grouping		
						Cont	3 min	10 min
*drat*	GB55016	Aggression/Hypoxia	Death resistor Adh domain containing target	16.1	**<0.0001**	**A**	**B**	**A**
*inos*	GB51125	Aggression	Inositol-3-phosphate synthase 1B	0.02	0.98			
*cyp6g1*/2*	GB52023	Aggression	Cytochrome P450	0.61	0.55			
*Unknown*	GB53860	Aggression	none	2.34	0.12			
*hif1α*	GB44532	Hypoxia	Hypoxia-inducible factor	5.47	**0.011**	**A**	**B**	**A**
*hsf*	GB52628	Hypoxia	Heat shock factor	4.03	**0.031**	**AB**	**A**	**B**

## Discussion

Here we find that smoke, which blocks olfactory cue perception during a lab-based aggression assay, temporarily inhibits aggression. Consistent with our hypothesis, this behavioral effect is accompanied by limited expression changes for aggression biomarker genes (only one of four aggression biomarker genes responded to smoke). In contrast, smoke exposure rapidly and temporarily increased brain expression of two hypoxia genes, suggesting a neuromolecular response to smoke that is not specific to aggression. These data support the hypothesis that modulation of social cue perception that causes a temporary change in behavioral phenotype may do so without additional changes in brain molecular state related to social modulation of aggression. However, these results should be interpreted cautiously due to the limited set of aggression and hypoxia genes assessed in the current study.

This is the first study to evaluate the behavioral effects of smoke using a lab-based assay of aggression (see ref.^37^ for other assays of “calming” scents). The results show a robust effect of smoke. There was a ~75% and ~50% decrease in aggressive behavior at the 3-min and 10-min time points, respectively, with a return to control aggression levels by 30 min. These patterns of behavioral change are evident for virtually all levels of aggressive behavior, from antennation to stinging. The time course of effect is in agreement with a previous study assessing the antennal response to alarm pheromone following exposure to smoke, which documented a decline in responsiveness that lasts at least 10 min^29^. This strong effect of smoke is well beyond the typical changes in behavior elicited by social cues^17,22,28,38^ or targeted pharmacological manipulations^30^, a result that is consistent with substantial blockage of the ability of worker bees to perceive aggression-inducing cues. Though workers may perceive visual, behavioral, and olfactory cues in this lab-based aggression assay, our results highlight the fact that the major cues that alert workers to the presence of a non-nestmate threat are olfactory: hydrocarbons present in the cuticle of the intruding bee signal a non-nestmate^32,39^. Thus, smoke appears to block odor perception in multiple contexts relevant to aggression: it impairs the perception of alarm pheromone, inhibiting the anti-predator response, as well as the perception of nestmate recognition odors, inhibiting the aggressive response towards an intruding conspecific (the current study).

Smoke exposure had relatively limited impacts on brain aggression biomarker expression, causing expression changes in only one of four genes. Previous studies using these genes show robust changes in at least 3 genes in association with behavioral variation (though notably expression was evaluated at a later time point in these studies^9,28^). Interestingly, the single differentially expressed biomarker gene in our study, the honey bee ortholog to the *Drosophila melanogaster* gene *drat*, shows inconsistent effects in contexts of socially modulated aggression^9,28^, and in our study, it is upregulated by smoke, despite its typical pattern of down-regulation in low aggression states^9^. In fruit flies, *drat* has been implicated as an important player in the hypoxia response: it is upregulated with chronic and intermittent hypoxia, and increased *drat* expression enhances survival following hypoxia treatment^40^. Similarly, here we see a strong increase in *drat* expression 3 min following smoke exposure, a pattern that matches expression of *hif1α* and *hsf*, and is suggestive of a role for this gene in the honey bee hypoxic response.

This dual role of *drat* in aggression and hypoxia is not necessarily surprising; many genes associated with social behavioral regulation are broadly involved in other general organismal processes^14,41^. Moreover, in honey bees, a 1-min exposure to alarm pheromone and exposure to an intruder bee both cause a rapid decline in oxygen-dependent metabolism measured at both physiological and molecular levels: neural mitochondrial respiration declines^31,42^, and there is decreased expression of genes associated with oxidative phosphorylation^23,27,30,43^. These changes in oxygen-dependent metabolic processes are not necessarily directly driven by limited oxygen availability to the brain in the context of aggression, but may instead reflect a change in neural energetic state that facilitates response to aggression-inducing cues^31,44^. *Drat* may be involved in or modulated by this process. Alternatively, a change in *drat* expression could reflect a more generalized response to stress^45^. Because there is evidence that stress, hypoxia, and aggression are phenotypically linked, one alternative interpretation of our results is that the degree of smoke exposure or the time points selected do not adequately capture the complexity of the molecular response.

In addition to causing a temporary decrease in aggression, smoke exposure has other behavioral effects, one of which is honey engorgement, where workers rapidly eat large amounts of honey^46^. The proposed adaptive significance of this behavior is that it allows bees to carry a greater proportion of their food stores with them as they abandon their hive during a tree fire^47^. However, this behavioral change may also be related to a shift in brain metabolism, specifically an increased demand for glucose^43,48^. Previous studies, for example, have shown that efficacy of smoke on aggressive behavior is inversely related to the quantity of honey consumption^46^, which may indicate that smoke influences the efficiency of olfactory inhibition and honey engorgement in a dose-dependent way. These patterns suggest that honey engorgement may be a physiological side effect of smoke exposure, and not an adaptive behavioral response *per se*.

Here we show that it is possible to manipulate aggressive behavior through odor perception without causing changes in the expression of genes strongly associated with aggression. These results emphasize the possibility that differential perception of cues can modulate behavior without causing further variation in brain molecular state^28^, particularly in cases where behavioral shifts are temporary. Overall, these findings support the behavioral genomics theory that at least some socially-induced changes in gene expression cause lasting behavioral change, and do not simply reflect neural activation and recovery. Our results may be highly generalizable, particularly to other social species, especially social insects, where aggression in the context of nestmate recognition is often mediated by odor cues^49–51^. Despite our small gene sets, the aggression biomarker genes in particular have been employed across many different experimental contexts yielding different results, providing a foundation to robustly interpret our current data^9,28^. The candidate gene approach presented here, while it may be relatively limited in physiological insight compared to whole-transcriptome sequencing, nonetheless provides a hypothesis-driven framework to identify and interpret gene expression dynamics and its links to behavioral expression in specific social contexts.

## Data Availability

Data will be made accessible upon request.
